# Custom-Made Artificial Iris and Toric-Intraocular Lens Intrascleral Flange Fixation: A Case Report

**DOI:** 10.3390/medicina60060865

**Published:** 2024-05-25

**Authors:** Ran Moshkovsky, Elinor Megiddo-Barnir, Guy Kleinmann

**Affiliations:** 1 Department of Ophthalmology, Wolfson Medical Center, Holon 5822012, Israel; emegiddo@gmail.com (E.M.-B.); guykl.email@gmail.com (G.K.); 2 School of Medicine, Faculty of Medical and Health Sciences, Tel Aviv University, Tel Aviv 6997801, Israel

**Keywords:** aniridia, aphakia, artificial iris, astigmatism, toric-intraocular lens

## Abstract

Different techniques for artificial iris implantation with or without an intraocular lens, depending on lens status, are described in the literature. We describe a surgical technique for a custom-made artificial iris and toric-intraocular lens intrascleral flange fixation. We modified the “Backpack” artificial iris implantation surgical technique to facilitate an accurate alignment of the toric-intraocular lens in a patient with aphakia, aniridia, and high asymmetric astigmatism secondary to blunt trauma. Two months after the surgery, uncorrected visual acuity was 20/30, corrected to 20/25 with a refraction of −2.00 in the diopter sphere with no residual astigmatism. The artificial iris implant and toric-intraocular lens were well-centered. The patient was satisfied with the visual and cosmetic outcomes. This procedure, however, is not complication-free as our patient developed uveitis and increased intraocular pressure during the postoperative period, which was treated successfully.

## 1. Introduction

Iris defects can be congenital or acquired. In traumatic cases, the extent of iris defects can range from traumatic mydriasis and partial iris loss to complete aniridia. The eye often has additional alterations, such as wound scarring, aphakia or a traumatic cataract, corneal astigmatism, and possibly retinal damage [1]. Colored contact lenses, corneal tattooing, or merely sunglasses are among the conservative treatments [2,3]. In the cases of iris defects that are limited to approximately two clock hours, reconstruction of the pupil using iris sutures is of consideration. The artificial iris (AI) is a relatively new solution [4]. The implant can be fixated using several techniques with or without intra-ocular lens (IOL) implantation. While there is a variety of existing techniques for AI implantation, the reported technique addresses the challenge of precise toric–IOL axis alignment when the IOL is fixated on the posterior aspect of the AI.

## 2. Material and Methods

A 42-year-old male patient had sustained traumatic penetration of the right eye globe due to blunt trauma. The patient had no previous medical or ocular history. An initial examination revealed a ruptured sclera with the loss of the iris diaphragm and crystalline lens. The patient underwent immediate primary closure of the globe followed by a pars-plana vitrectomy with laser barrage 2 weeks later due to a vitreous hemorrhage. At one month, uncorrected visual acuity (UCVA) was one-meter finger count, corrected to 20/20-partial with a refraction of +10.00 D/−2.25 D×65°. On biometry, a +4.05 D @ 165° astigmatism was measured compared to only +0.59 D @ 133° in the other eye. Tomography showed a regular bow-tie astigmatism of +3.78 D @ 167° compatible with the biometry values. In addition to being aphakic, the patient also suffered from photophobia and glare secondary to the aniridia. After a discussion with the patient, a toric-intraocular lens (IOL) correction was chosen, along with an AI implant. The following surgery was conducted ten months after the primary repair to allow complete wound healing. Biometry and tomography measurements remained stable.

**Figure 1:**
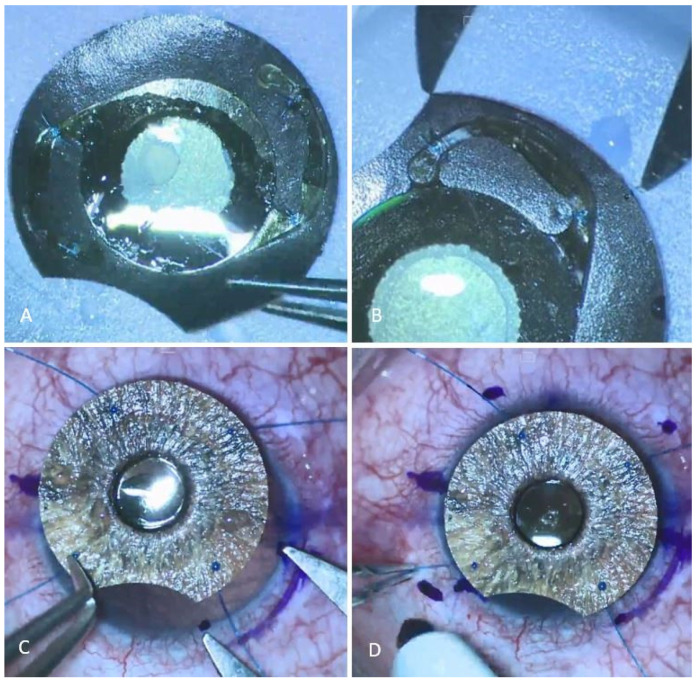
Toric–IOL is fixed to the back of the AI implant with sutures at the optic–haptic junction and at each distal end of the haptics (**A**). Measuring the distance between the IOL axis to the edge of the iridectomy (**B**). Marking the distance between the IOL axis to the edges of the iridectomy on the sclera (**C**). Marking the locations of the four flanges on the sclera in accordance with the iridectomy marks (**D**).

The process for assembling an AI implant-toric–IOL complex is as follows: A trephine was used to cut the customized AI implant (Customflex^®^, HumanOptics, Erlangen, Germany) according to the patient’s eye white-to-white measurements followed by iridectomy (Supplemental Video S1). A small amount of the cohesive ophthalmic viscosurgical device (OVD, Biolon^®^, Bio-Technology General Ltd., Be’er Tuvia, Israel) was placed on the back surface of the AI implant to facilitate the stable placement of a one-piece toric–IOL prior to suturing: First, the IOL, Acrysof^®^, IQ Toric SN6AT9 +18.00 Diopter, (Alcon, Vernier, Switzerland) was fixated to the AI implant using 10-0 polypropylene sutures (PROLENE^®^, Ethicon LLC, San Lorenzo, PR, USA) that ran within the iris implant material at the optic–haptic junction, keeping it discrete from the front surface of the AI, and around the IOL haptics on both sides. The IOL was set on the AI implant so that the iridectomy could be positioned superiorly. Second, each suture was threaded beneath its haptic to form another knot, tying the suture to the haptic itself, thereby preventing slippage. In the same manner, another suture was added to each distal end of the haptics so that the haptics’ edge was restricted from chafing the ciliary body (Figure 1A). 

Figure 1, marking the toric–IOL axis: The axis was marked at the AI rim in accordance with the manufacturers’ imprinted three-point axis markings of the IOL. The distance between the mark made on the AI rim to the edge of the iridectomy was then measured for later reference on both sides (Figure 1B).

The intra-scleral flange fixation of the AI implant-toric–IOL complex: A 27-G needle was used to pierce through the peripheral posterior surface of the AI implant, and a 6-0 polypropylene suture (PROLENE^®^, Ethicon LLC, San Lorenzo, PR, USA) was threaded into the needle and through the implant. A flange was created at the anterior surface of the implant using a low-temperature cautery (Kirwan Surgical Products LLC, Marshfield, MA, USA). The same procedure was repeated for the remaining quadrants of the AI. 

The location of the main incisions and the IOL axis were marked on the eye according to the 0-, 180-, and 270-degree pre-marks that were performed while the patient was in a sitting position. The correct location for placing the iridectomy edges (implicating the correct axis position) was marked using the reference distance measured earlier, the distance between the IOL axis, and the edges of the iridectomy on both sides (Figure 1C). The implant was then placed on the cornea according to the iridectomy marks and the locations for the four flanges were marked on the sclera, about 1.5 mm posterior to the limbus (Figure 1D). After peritomy, a superior 3 mm scleral tunnel was created using a crescent knife and a keratome (MANI, Inc., Tochigi, Japan). Four bent 27-G needles were passed through scleral tunnels into the eye at the 4 locations of the scleral marks for proper AI placement to ensure toric IOL alignment. The 6-0 polypropylene sutures were then introduced into the eye through the main incision and carefully directed into their respective 27-G needles to be led outside the eye. Primary flanges were created at the tip of each suture. The entire AI implant-toric–IOL complex was folded using forceps and inserted into the eye. After the complex was well centered inside the eye, the scleral tunnel was sutured using a 10-0 nylon suture (ETHILON^®^, Ethicon LLC, San Lorenzo, PR, USA), and the fixating sutures were gently tensioned and trimmed, creating new flanges.

## 3. Results

Two weeks after the surgery, the patient developed anterior uveitis with increased intraocular pressure (IOP), which was treated with topical and oral steroids with the resolution of the inflammatory response. The increased IOP did not respond to maximal medical therapy, and a filtration device (PRESERFLO™ MicroShunt, InnFocus, Inc., Miami, FL, USA) was placed successfully.

**Figure 2:**
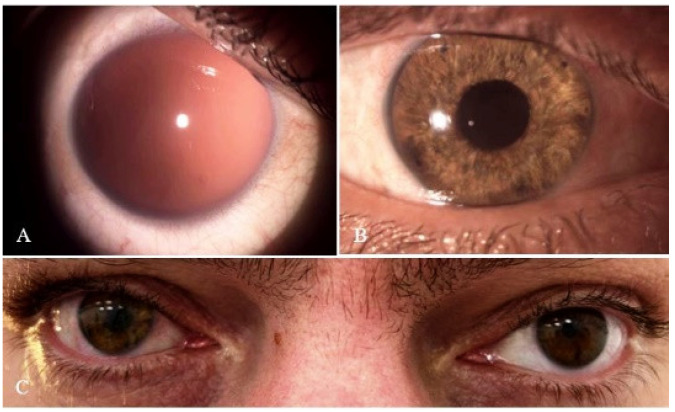
Preoperative (**A**) and two-month postoperative (**B**,**C**) images after implantation of the AI with toric–IOL in the right eye.

Two months after the surgery, UCVA was 20/30, corrected to 20/25 with a refraction of −2.00D in the sphere with no residual astigmatism. IOP was 9 mmHg with an elevated active bleb, deep and quiet anterior chamber, and the AI implant and toric–IOL were well-centered. The patient was satisfied with the visual and cosmetic outcomes (Figure 2).

Figure 2: ten months after the surgery, the patient developed localized corneal edema due to the proximity of the shunt to the cornea, and the shunt was removed. Target IOP was maintained using medical therapy alone until the last follow-up visit, four months after shunt removal.

## 4. Discussion

The rehabilitation of an aphakic, aniridic eye due to penetrating trauma is challenging. It is an even greater challenge when there is a high asymmetric corneal astigmatism in such an eye, requiring the proper alignment of toric–IOL. The described technique provided good functional outcomes and a cosmetically appealing result while addressing these challenges. The procedure is not complication-free. Complications include elevated IOP, secondary glaucoma, persistent inflammation, retinal detachment, and corneal decompensation [5,6,7]. An elevated IOP is the most frequent adverse effect after AI implantations, which is often due to severe alterations in the globe caused by trauma, anterior synechia, or narrow angles [5]. During the postoperative period, our patient developed uveitis, which was controlled with topical steroid treatment and elevated IOP, requiring filtration surgery. In this case, the mechanism for the high IOP was probably attributed to the uveitis. Angle-closure is less likely, as an iridectomy was performed on the AI implant. Also, a small crescent-shaped space remained between the temporal edge of the implant and temporal angle structures (Figure 2B). It is possible that the sustained control of the postoperative inflammatory response followed by the resolution of trabeculitis allowed for partial recovery of the trabecular meshwork architecture and function, enabling the patient to maintain target IOP using medical therapy alone after shunt removal. 

The patient had no previous medical history, but one should bear in mind that acute anterior uveitis could manifest as a systemic disease. For example, ankylosing spondylitis (AS), typically occurring among young men, is a critical etiology of acute anterior uveitis. Uveitis is the most common extra-articular manifestation of AS, and prompt treatment with steroid-sparing agents should be issued in such cases so as to prevent glaucoma [8]. 

The mainstay of the presented technique is the “Sandwich” or “Backpack” AI implantation in which the IOL is sutured to the back of the AI using the IOL haptics and inserted into the eye as a folded sandwich [9]. The advantages of this technique are the small surgical incision due to a foldable AI–IOL package and minimal additional trauma, as most of the surgery takes place outside the eye. In addition, to attain a suture-less scleral fixation of the AI–IOL complex, we adopted the flanged fixation technique developed by Canabrava et al. [10]. In this regard, it is noteworthy to keep in mind the risk of postoperative endophthalmitis secondary to flange erosion or extrusion and implement the appropriate steps for flange creation and coverage [11]. 

## 5. Conclusions

We present a technique for anatomic and visual rehabilitation in the case of apakia, aniridia, and significant asymmetric corneal astigmatism, which facilitates the exact alignment of a toric-intraocular lens along with a custom-made artificial iris. Nevertheless, this procedure is not complication-free, and this may impair its long-term effectiveness.

## Data Availability

No new data were created or analyzed in this study.

## Supplementary Materials

The following supporting information can be downloaded at: https://www.mdpi.com/article/10.3390/medicina60060865/s1, Video S1: Assembling an AI implant toric–IOL complex, marking the toric–IOL axis and intra-scleral flange fixation of the AI implant toric–IOL complex.
